# Role of the CASZ1 transcription factor in tissue development and disease

**DOI:** 10.1186/s40001-023-01548-y

**Published:** 2023-12-05

**Authors:** Tiantian Liu, Tao Li, Shaorui Ke

**Affiliations:** 1 Henan Key Laboratory of Chinese Medicine for Respiratory Disease, Henan University of Chinese Medicine, 156 Jinshui East Road, Zhengzhou, 450046 Henan China; 2 Academy of Chinese Medical Sciences, Henan University of Chinese Medicine, Zhengzhou, 450046 Henan China; 3 College of Life Sciences, Henan Agricultural University, Zhengzhou, 450002 China

**Keywords:** CASZ1, Development, Biomarker, Cancer

## Abstract

The zinc finger transcription factor gene, *CASZ1/Castor* (Castor zinc finger 1), initially identified in *Drosophila*, plays a critical role in neural, cardiac, and cardiovascular development, exerting a complex, multifaceted influence on cell fate and tissue morphogenesis. During neurogenesis, CASZ1 exhibits dynamic expression from early embryonic development to the perinatal period, constituting a key regulator in this process. Additionally, CASZ1 controls the transition between neurogenesis and gliomagenesis. During human cardiovascular system development, CASZ1 is essential for cardiomyocyte differentiation, cardiac morphogenesis, and vascular morphology homeostasis and formation. The deletion or inactivation of CASZ1 mutations can lead to human developmental diseases or tumors, including congenital heart disease, cardiovascular disease, and neuroblastoma. CASZ1 can be used as a biomarker for disease prevention and diagnosis as well as a prognostic indicator for cancer. This review explores the unique functions of CASZ1 in tissue morphogenesis and associated diseases, offering new insights for elucidating the molecular mechanisms underlying diseases and identifying potential therapeutic targets for disease prevention and treatment.

## Introduction

The *CASZ1/Castor* (Castor zinc finger 1) gene, initially discovered in *Drosophila*, encodes a zinc finger transcription factor (TF) that regulates neural fate [1]. In humans, CASZ1 is located on chromosome 1p36, acting as a tumor suppressor gene. It encodes two primary isoforms: CASZ1a, spanning 1759 amino acids with 11 TFIIIA-like C2H2 zinc fingers (ZnFs), and CASZ1b, a more evolutionarily conserved isoform containing 1166 amino acids and lacking 6 zinc fingers in the C-terminal region [2]. Highly conserved noncoding DNA elements that are present in the noncoding region of the *CASZ1* gene across species from *Drosophila* to humans [3–5] are strongly associated with developmentally regulated genes [5, 6]. In *Drosophila*, *Casz1* functions as a neuronal fate-determining gene, controlling nervous system development [1, 7, 8], and its loss affects differentiation and alters glial cell numbers and migration [1, 7–9]. In *Xenopus laevis* and humans, CASZ1 plays a critical role in heart development, cardiomyocyte differentiation, vascular assembly, and lumen morphogenesis [10–12].

In addition to its crucial role in both neural and cardiac development, the involvement of CASZ1 in various pathological conditions, including cancer, is being increasingly recognized [13]. In cancer, CASZ1 has emerged as a key player in tumor progression, regulating tumor growth and development. The loss of heterozygosity and/or EZH2-mediated H3K27me3 modification can silence the *CASZ1* gene in tumor samples from patients with a poor prognosis of neuroblastoma (NB) [14]. A clinical case report showed human papillomavirus DNA integration into the *CASZ1* gene locus in a patient with cervical cancer, which disrupted *CASZ1* gene expression [15]. Furthermore, a CASZ1–MASP2 fusion transcript, detected in colorectal cancer, encodes an N-terminally truncated MASP2 controlled by the CASZ1 promoter [16]. Similarly, CASZ1 exhibits downregulation in esophageal carcinoma, lung adenocarcinoma, and clear cell renal cell carcinoma, where expression levels correlate with patient prognosis [17–19]. However, the expression trend of CASZ1 varies in different solid tumors, exhibiting upregulation in glioma tissues and epithelial ovarian cancer (EOC) cells [20, 21]. Therefore, CASZ1 plays dual biological roles in diverse tumors. Beyond cancer, loss-of-function mutations in CASZ1 have been associated with susceptibility to human heart diseases [22–25], and CASZ1 methylation has been associated with cardiovascular mortality [26]. Additionally, CASZ1 has been implicated in osteoarthritis, immune inflammatory, and regulatory responses [27].

This review provides a comprehensive summary of the role of CASZ1 in both development and disease, offering novel insights into the molecular mechanisms underlying various diseases. Moreover, it highlights the potential of CASZ1 as a prognostic indicator and therapeutic target for diagnosing and treating these diseases, thereby contributing to the advancement of medical research.

## Discovery and structural features of CASZ1

In the 1990s, an enhancer detection screen identified a new gene, *Castor*, which is required for the development of the central nervous system in *Drosophila* embryos [1]. The Castor zinc finger protein is expressed in subsets of the Drosophila ventral nerve chord and the procephalic region during embryogenesis. Loss of Castor function leads to precise changes in gene expression in the central nervous system, as well as defects in axonogenesis and embryonic lethality [1, 28–31].

The expression of Castor in *Drosophila* at a developmental stage comparable to later stages of human embryonic neurogenesis [32], led to the identification and cloning of the highly expressed human CASZ1 gene. CASZ1 is expressed in various human tissues, including the heart, lung, skeletal muscle, pancreas, testis, small intestine and stomach, but not in the adult brain [2]. The CASZ1 gene has two mRNA isoforms, hCasz5 (CASZ1b) and hCasz11 (CASZ1a), which are 4.4 kb and 8.0 kb in length, respectively. The hCasz5 isoform encodes a protein of 1166 amino acids (127.7 kDa, p*I* 8.4), containing five TFIIIA class C2H2 ZnF motifs. In addition, the hCasz5 isoform has two nuclear localization signals (NLS) located at 23–29 and 232–248, a nuclear export signal (NES) located at 168–186 [33], a nucleosome remodeling and deacetylase (NuRD) complex binding site located at 21–45, a histone H3 and DNA repair protein binding site located at 640–650 [34], a serine-enriched region located at 743–784, and a congenital heart disease 5 protein (CHD5)-interacting domain (CID) region located at 785–998 [24].

**Fig. 1:**
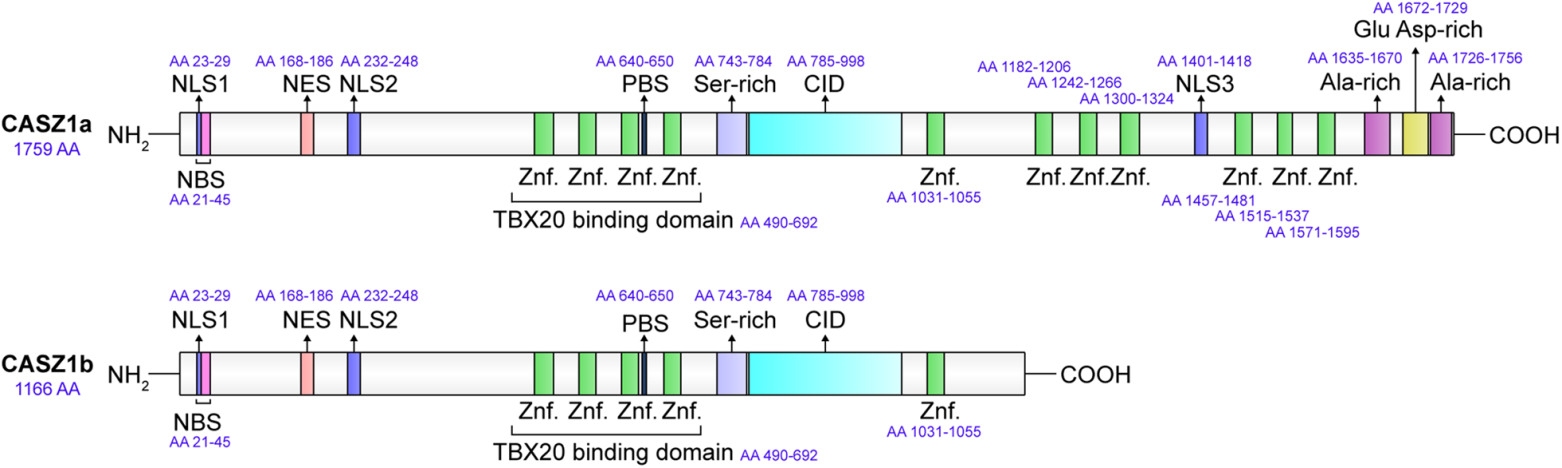
The protein structure of CASZ1. *Znf.* zinc finger, *NLS* nuclear localization signals, *NES* nuclear export signals, *NBS* NuRD complex binding site, *PBS* histone H3 and DNA repair protein binding site, *CID* CHD5-interacting domain

The cDNA sequence of CASZ1a is 7946 bp in length and codes for a protein with 1759 amino acids (190.0 kDa, p*I* 6.64). In addition to the information contained in CASZ1b, CASZ1a has six additional TFIIIA class C2H2 ZnF motifs. Furthermore, CASZ1a has one extra NLS located in 1401–1418, one Glu Asp-rich region located in 1672–1729, and two Ala-rich regions located in 1635–1670 and 1726–1756 [2]. The protein structure of CASZ1 is presented in Fig. 1.

## In neural development

Neural progenitor cells undergo identity transitions, generating diverse neurons and glial cells in a precise manner during development. Throughout neurogenesis, the majority of neural stem and progenitor cells alter their output, initially producing neurons followed by glial cells [35]. The occurrence of different cortical cell populations is time-separated; in rodents, neurons are produced from embryonic day 12 (E12) to E18, astrocytes appear around E18, and their numbers reach a peak in the neonatal period. Differentiated oligodendrocytes first appear after birth [35]. In vitro primary E10–E12 cortical precursor cells only generate neurons in the first few days, followed by astrocytes and oligodendrocytes [36, 37]. In addition, when the very early cortex is transduced, many precursors only produce neurons, some precursors produce both neurons and glial cells. Surprisingly, some precursors only produce glial cells, which may be because they wait until a later time point to differentiate. Therefore, there are two major conclusions for these studies. First, the capacity of precursor cells changes over time, with a preference for making neurons earlier and glia later. Second, the external environment of the precursor is a key determinant of its differentiation [35]. Certain central nervous system (CNS) regions, such as the neocortex and retina, exhibit additional temporal transitions in progenitor cells at specific developmental stages, generating distinct neuronal subtypes. For instance, during vertebrate retinal development, retinal progenitor cells (RPCs) generate various neuronal subtypes and glia at different pluripotent stages [38–40]. In the *Drosophila* ventral spinal cord, neural stem cells express TFs, including hunchback, Krüppel, nub/pdm2 (collectively pdm), castor, and grainyhead, during development. These temporal recognition factor cascades act as a timing mechanism, coordinating the output of numerous neuroblast lineages within the CNS of *Drosophila* [41].

Casz1 is expressed in the dorsal root ganglia and spinal cord of mice during neurogenesis [11, 42, 43], displaying ubiquitous expression from early embryonic development to the perinatal period. Initially expressed in dorsal interneuron 1 progenitors and their derived neurons in the dorsal spinal cord, Casz1 later extends to a large subset of dorsal late-born excitatory (dILB) neurons. Prrxl1, a key TF for dILB differentiation, positively regulates Casz1 expression in the dorsal embryonic spinal cord. During the perinatal period, Casz1 expression is maintained in a narrow cell layer, predominantly within layer III of the dorsal horn independent of Prrxl1 [44]. Both mouse CASZ1a (mCASZ1a) and CASZ1b (mCASZ1b) exhibit dynamic expression in tested neural tissues, with the expression pattern regulated according to the isoforms ratio [45]. Furthermore, neural differentiation during development is often accompanied by prolonged cell cycles [46, 47], with CASZ1-induced changes potentially extending the cell doubling time, thereby serving as a potential mechanism for neuronal differentiation regulation.

## In retinal development

In mouse retinal progenitors, similar to *Drosophila* neuroblasts, Casz1 regulates temporal progression through a conserved transcriptional cascade. Additionally, Casz1 plays a crucial role in regulating progenitor cell potential and controlling the generation of mid/late-born neurons in the mouse retina. Predominantly observed in middle and late RPCs, conditional Casz1 loss enhances early retinal neuron generation at the expense of late fates [42]. Furthermore, Casz1 interacts with key polycomb repressive complex (PRC) subunits, controlling rod genome organization by silencing laminin a/c [48]. In addition, it epigenetically regulates transcriptional programs by binding to the NuRD complex in retinal cells [34], relying on the NuRD complex and PRC to promote rod fate while suppressing gliogenesis [49].

## In heart development

Early heart development is characterized by hyperplastic growth, wherein cardiac cells undergo mitogen-dependent activation during the G1 phase of the cell cycle [50]. During initial heart development stages, cardiomyocytes from the first and second heart fields exhibit high proliferative activity, contributing to substantial embryonic heart growth. As cardiomyocyte terminal differentiation commences, the overall cardiomyocyte proliferation rate gradually diminishes. Subsequently, the vertebrate heart primarily enlarges through hypertrophy, cellular recruitment, and proliferation of the neural crest and epicardium [51], with these processes sustaining heart development and growth.

In humans, CASZ1 exhibits high expression levels in various organs, including the heart, lungs, skeletal muscle, pancreas, testes, small intestine, and stomach. Notably, the highest relative expression level of CASZ1 is observed in the heart [2] where it plays a crucial role in morphogenesis and development [11, 52]. Specifically, during development, Casz1 is vital for the differentiation of distinct cardiomyocytes, exhibiting continuous expression throughout cardiac development. Furthermore, CASZ1 is exclusively expressed in terminally differentiated cardiomyocytes and downregulated in cells re-entering the cell cycle, indicating its association with the terminal differentiation of cardiomyocytes, skeletal muscle cells, and lymphatic cardiac muscle tissue [43]. Moreover, CASZ1 is critical for cardiomyocyte proliferation in two heart regions during the earliest stages of mammalian heart development. CASZ1 loss results in a reduced number of cardiomyocytes, prolonged or arrested S phase, decreased DNA synthesis, increased phosphorylated RB, and decreased cardiac mitosis [51]. In *Xenopus* embryos, CASZ1 deficiency leads to the failure of ventral midline progenitors to differentiate into cardiomyocytes, resulting in abnormal cardiac morphogenesis and death [10]. Furthermore, the abnormal expression of CASZ1 target genes, such as muscle contractile genes (*TNNI2*, *TNNT1*, and *CKM*), contractile fiber genes (*ACTA1*), and genes encoding arrhythmia-associated ion channels (*ABCC9* and *CACNA1D*) occurs in Casz1-null mouse embryonic hearts, leading to myocardial hypoplasia and congenital ventricular septal defects (VSDs) [11]. Additionally, CASTOR (CASZ1) interacts with congenital heart disease 5 protein (CHD5) or TBX20, a necessary interaction for cardiac morphogenesis and homeostasis [52, 53].

## In cardiovascular development

During embryonic development, endothelial cells (ECs) serve as the foundation for functional vasculature formation. The initial phases involve vasculogenesis, where mesodermal cells differentiate into EC progenitors, which subsequently proliferate and migrate, assembling into vascular cords at specific embryo sites. Then, the umbilical cords undergo tubulogenesis or lumen formation and mature through angiogenesis, which involves vessel budding, branching, and remodeling. Finally, pericytes and smooth muscle cells provide structural support by surrounding and stabilizing the vessels [54, 55].

**Fig. 2:**
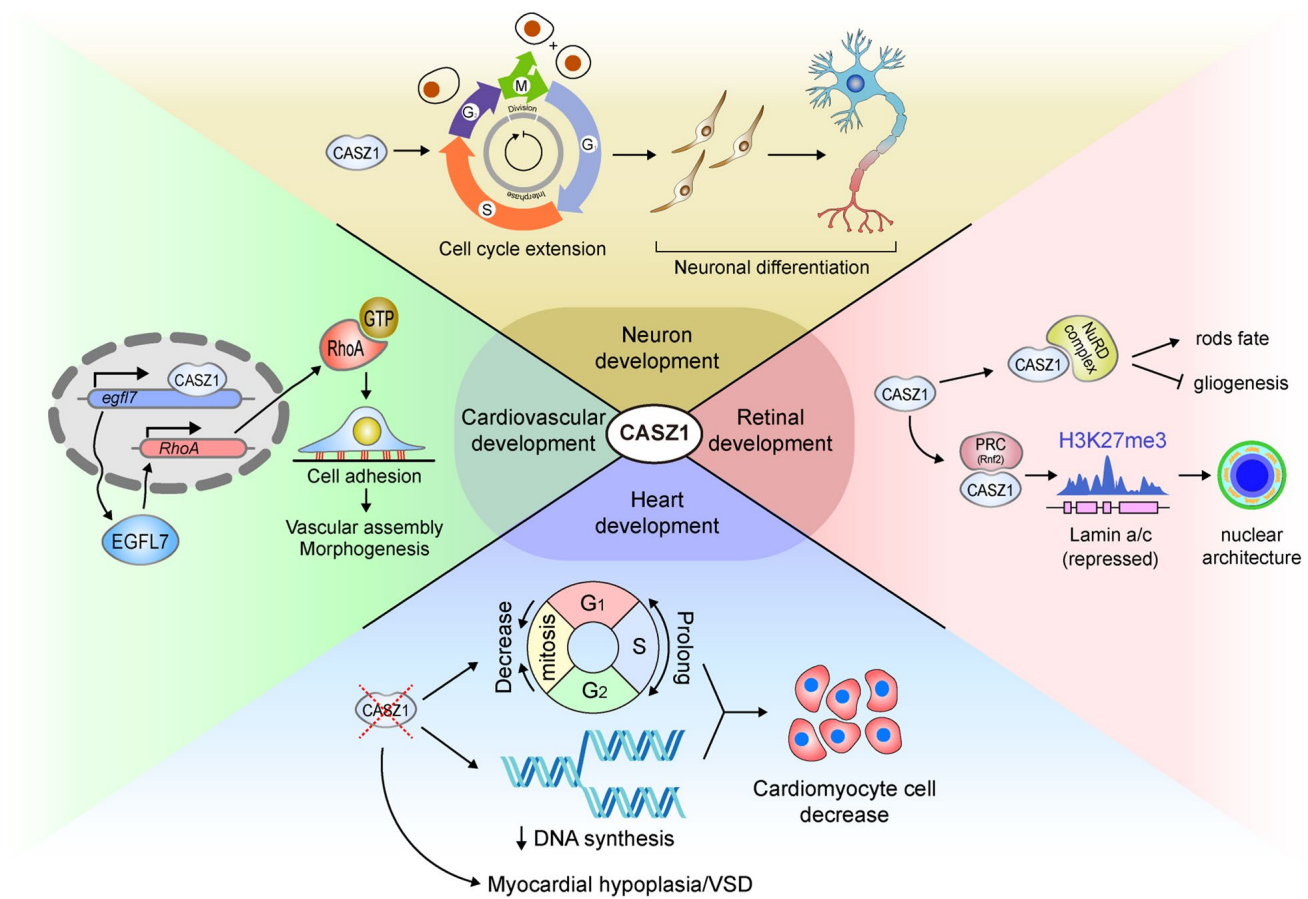
The role of CASZ1 in tissue development: CASZ1 plays a crucial role in the development of various tissues. It regulates the development of the nervous system and facilitates the production of retinal neurons. Additionally, CASZ1 is essential for the differentiation of myocardial cells and the maintenance of cardiac morphogenesis and homeostasis, as well as the formation of vascular morphology

Two genome-wide association studies (GWASs) have revealed a genetic association between the human *Casz1* locus and hypertension [56, 57], suggesting a potential link between CASZ1 and cardiovascular dysfunction. The epidermal growth factor-like domain 7 (*EGFL7*) gene, activated by Castor (CASZ1), is crucial for vessel assembly and luminal morphogenesis [12, 58, 59]. In *Xenopus* and human epithelial cells, CASZ1 directly regulates Egfl7, thereby promoting RhoA-mediated vascular development in vertebrates. CASZ1 deficiency not only impedes the branching and luminal vasculature development of *Xenopus* embryos, but also induces marked changes in human EC adhesion, morphology, and sprouting [12, 59]. The mechanisms of CASZ1 regarding tissue development are summarized in Table 1 and Fig. 2.

**Table 1:** Potential role of CASZ1 in tissue development

Tissue development	Cell types involved	Proposed mechanism	Refs.
Nerve	Dorsal root ganglia and spinal cord	Ratio between casz1a/casz1b isoforms	[45]
Retina	Retinal progenitor cells	Interacts with PRC or NuRD complex	[41–43]
Heart	Cardiomyocytes	Regulates cell cycle or interacts with CHD5/TBX20	[51–53]
Angiography	Endothelial cells	Regulates the Egfl7-RhoA pathway	[12, 59]

## In NB

NB is a prevalent solid tumor that affects the pediatric population, predominantly targeting the brain and accounting for ~ 15% of all pediatric tumor-related deaths, and has a poor prognosis [60]. The CASZ1 TF, a zinc finger protein, plays a critical role in nerve cell development regulation. Abnormal CASZ1 expression has been implicated in the malignant behavior of human NB [61]. Notably, both CASZ1a and CASZ1b isoforms have been identified as inhibitors of NB tumor growth [2, 45, 61, 62]. CASZ1b is expressed in all CASZ1a-expressing NB samples and mouse tissues. Throughout the differentiation of neuroblasts and myoblasts, both the CASZ1 isoforms exhibit coordinated regulation, displaying temporal and region-specific regulation during neurogenesis in vivo. Functionally, CASZ1b and CASZ1a exhibit similar gene-regulatory activities, with their coexpression showing no cross-antagonistic or synergistic effects [45].

The loss of CASZ1 in NB tumors correlates with poor prognosis. An examination of CASZ1 protein expression in a primary NB tissue microarray revealed that tumors from patients with NB and favorable prognoses exhibited higher levels of nuclear CASZ1 protein expression. Conversely, tumors from patients with unfavorable prognosis exhibited cytoplasmic-restricted CASZ1 staining or low nuclear CASZ1 staining [33]. Furthermore, the growth-inhibitory capacity of cytoplasmic-localized CASZ1b is significantly reduced [62]. CASZ1 expression levels increase with the induced differentiation of NB cells and mesenchymal cells [2]. Additionally, low CASZ1 expression exhibits a significant association with increasing patient age, high-risk classification of pediatric tumor group, loss of heterozygosity on chromosome 1p (1p LOH), MYCN amplification, and reduced survival probability. CASZ1 (1p36.22) is located in a common deletion region marked between D1S508 and D1S244 on chr1p. In a study involving 184 primary NB cases with 1p LOH, 180 cases showed CASZ1 deletion. The specific restoration of CASZ1 in NB cells induces cell differentiation, enhances cell adhesion, inhibits migration, and reduces tumorigenicity [61, 62]. No tumor-associated nucleotide mutations have been reported in the CASZ1-coding sequence, suggesting that mechanisms such as epigenetic silencing may be associated with reduced CASZ1 expression in tumors from patients with poor prognosis [63, 64].

Histone 3 trimethylation at lysine 27 (H3K27me3), a histone modification associated with gene silencing, is catalyzed by the methyltransferase EZH2, an enzymatically active component of the PRC2 [65]. EZH2 is highly expressed in undifferentiated or poorly differentiated stromal NB tumors, and its overexpression correlates with poor prognosis. NB tumor suppressor genes, including *CASZ1*, *CLU*, *NGFR*, and *RUNX3*, are direct targets of EZH2- and H3K27me3-mediated gene silencing [14]. Knocking down EZH2 expression using RNA interference or inhibiting its expression using 3-deazaneplanocin A drugs increases CASZ1 expression, inhibits NB cell growth, and induces neurite extension [14].

Although demethylating agents can induce CASZ1 expression, the methylation status of the 5ʹ and 3ʹ CpG-rich regions of the *CASZ1* gene, determined via bisulfite sequencing, appears insufficient to explain the low CASZ1 expression level [61, 63]. HDAC inhibitors, such as depsipeptide, can upregulate the expression of several genes, with TSA being the sole inducer of CASZ1 expression and three other genes among the 30 genes located in the shortest region of the overlap of Chr1p36 between markers D1S508 and D1S244 [66]. This suggests that only a subset of genes on Chr1p36 is silenced by histone deacetylation. Epigenetic silencing, whether direct or indirect, likely contributes to the low CASZ1 expression level in NB cells.

The dysregulation of the cell cycle mechanism marks cancer progression [67, 68]. Previous microarray analysis has revealed that tumor transcriptomes from patients with poor prognosis are enriched with cell cycle-related genes, whereas those from patients with good prognosis are enriched with differentiation-related genes [69]. Low CASZ1 expression, resulting from the loss of heterozygosity or epigenetic repression, is associated with abnormal regulation of cell cycle genes, including *Cyclin D1* and *Chk1*, leading to an undifferentiated NB phenotype. The increased expression of cyclin D1 and enhanced cyclin D-dependent kinase activity contribute to pRb hyperphosphorylation and E2F release, thereby activating E2F-dependent gene transcription and promoting cell cycle progression in the G1-S phase [70, 71]. CASZ1 restoration activates pRb in the G1 phase, suppressing the expression of G2/M regulators, Cyclin B1, and Chk1, thereby leading to prolonged cell cycle progression and decreased cell proliferation in NB [72]. Furthermore, neural crest lineage-regulated transcription factors constitute a core regulatory circuit (CRC) in NB to specify a noradrenergic tumor phenotype. In NB tumor cells, the CASZ1 tumor suppressor is silenced by the NB CRC component HAND2, whereas CRC components are highly expressed. Restored CASZ1 forms a negative feedback regulatory circuit with the established NB CRC, inducing noradrenergic neuronal differentiation in NB [73].

## In other tumors

CASZ1 exhibits distinct expression patterns across various solid tumors, manifesting dual biological functions contingent on tumor type. For instance, Wang et al. reported reduced CASZ1 expression in hepatocellular carcinoma tissues, which hindered abnormal tumor cell proliferation by modulating the MAPK/ERK signaling pathway alongside MMP2 and MMP9 expression in vitro [74]. Similarly, CASZ1 downregulation has been observed in colorectal cancer, esophageal cancer, lung adenocarcinoma, and clear cell renal cell carcinoma, where it has been associated with patient prognosis and could serve as a novel prognostic marker [17–19, 75]. Notably, the loss of CASZ1 activity can impede embryonal rhabdomyosarcoma differentiation through RAS-MEK signaling or genetic mutations, culminating in RMS tumor development [76].

Conversely, contrary to the that reported by several previous studies, high CASZ1 expression has been observed in glioma tissues, where it functions as an oncogene by regulating the transcription of its target gene *p75NTR* [20]. Additionally, CASZ1 is upregulated in EOC cells, promoting their epithelial–mesenchymal transition, whereas CASZ1 knockdown inhibits cancer cell metastasis in vivo [21]. Furthermore, in lung adenocarcinoma and idiopathic pulmonary fibrosis, CASZ1 shows hypermethylation and low expression, which is significantly associated with the prognosis of lung adenocarcinoma [18, 77]. Recent studies have shown that CASZ1 also plays an oncogenic role in lung cancer, with its expression positively correlated with cancer metastasis and poor prognosis. Specifically, CASZ1 regulates ITGAV expression, promoting lung cancer migration, invasion, and epithelial–mesenchymal transition [78]. The complex role of CASZ1 in malignant tumors may be related to tissue and tumor specificity. Hence, investigating the prognostic value of CASZ1 as a biomarker for cancer diagnosis and prognosis is imperative.

## In other diseases

In humans, the *CASZ1* gene is located on chromosome 1p36, and 1p36 deletion is the most prevalent telomere deletion. This deletion is causally related to congenital cardiovascular malformations and cardiomyopathy, which are the most common phenotypes of 1p36 deletion syndrome [79]. Specific missense (p.L38P) and nonsense (p.K351X) mutations have been identified in families with congenital VSD [22] and dilated cardiomyopathy (DCM) [23], respectively. Functional studies have revealed that the L38P and K351X mutant CASZ1 proteins lose their transcriptional activity. Additionally, a novel variant of the *CASZ1* gene, c.2443_2459delGTGGGCACCCCCAGCCT (p.Val815Profs*14), was identified in a patient with DCM and left ventricular noncompaction cardiomyopathy (LVNC), highlighting the role of *CASZ1* as a pathogenic gene for human LVNC [24]. In another case of DCM, a de novo frameshift mutation, c.3781del (p.(Trp1261GlyfsTer29)), was identified in the *CASZ1* gene [25]. Associating *CASZ1* loss-of-function mutations with human cardiac disease susceptibility holds potential implications for the personalized prevention and treatment of cardiac diseases.

An epigenome-wide association study, leveraging genome-wide transcriptome data, has revealed that CASZ1 methylation may serve as a regulatory element linked to mortality in patients with cardiovascular disease [26]. GWASs have established significant associations between DNA methylation and the risk of blood pressure (BP) and ischemic stroke, and CASZ1 was reportedly hypomethylated in Chinese patients with hypertensive cerebral infarction [80]. Additionally, CASZ1b, a newly discovered corepressor of the mineralocorticoid receptor (MR), is co-expressed with MR in MR target cells, including renal tubular cells. CASZ1b inhibits MR transcriptional activity, serving as an aldosterone-dependent adapter protein linking MR and the nucleosome remodeling deacetylase (Mi-2/NuRD) complexes, thereby inhibiting epithelial Na^+^ channel-α and serum/glucocorticoid-regulated kinase 1 expression, which ultimately lowers BP levels [81]. Nevertheless, injecting CASZ1 siRNA into mouse kidneys did not significantly alter BP [82]. In a recent GWAS study on primary aldosteronism, CASZ1 was identified as a gene associated with this condition, and the overexpression of CASZ1 inhibited aldosterone biosynthesis in adrenal cells [83]. These findings suggest that CASZ1 regulates hypertension and primary aldosteronism through dual mechanisms, namely, the modulation of MR transcriptional activity and aldosterone biosynthesis. GWAS on hypertension have reported that three single-nucleotide polymorphisms (SNPs) in the CASZ1 gene, rs880315 [57, 84, 85], rs284277 [56] and rs12046278 [86], are associated with hypertension.

Osteoarthritis is an age-related condition characterized by articular cartilage degeneration and joint inflammation that has garnered research attention in recent years. Notably, CircANKRD36 has emerged a key player in preventing chondrocyte apoptosis and counteracting inflammatory responses induced by IL-1β treatment. This protective effect is attributed to the ability CircANKRD36 to target miR-599, leading to the upregulation of Casz1 expression [27]. Casz1, a recognized regulator of T helper (Th) cell plasticity, holds major clinical relevance in autoimmune inflammation and mucosal immunity. In both in vitro and in vivo settings, Casz1 plays a vital role in Th differentiation, as evidenced by the reduced susceptibility of CD4^+^ T cells lacking Casz1 to experimental autoimmune encephalomyelitis. Furthermore, the loss of Casz1 results in the severe impairment of Th17 and Treg responses during mucosal *Candida* infection, rendering mice deficient in Casz1 less capable of clearing secondary infections [87].

Genome-wide DNA methylation analysis of whole blood from monozygotic twins has revealed that CASZ1 DNA methylation variants are negatively associated with fasting plasma glucose (FPG) levels [88]. Abnormal DNA methylation levels in the promoter region of the placental CASZ1 gene may lead to metabolic diseases including type 2 diabetes mellitus (T2DM) [89]. The association between CASZ1 gene variants and stroke risk in Chinese population studies have shown that CASZ1 genetic variants rs4845941 and rs778228 are significantly associated with an increased risk of stroke. Gender-stratified analysis also shows that CASZ1 rs778228 locus is associated with a higher risk of stroke in females. CASZ1 and its related genes may promote the occurrence of stroke, which is of great significance for the treatment and prevention of stroke [90]. There is a reproducible association between rs11121615 SNP, located within an intron of CASZ1 gene, and chronic venous disease (CVD) [91–93]. In addition, the mutation frequency of SNPs rs10511083 of CASZ1 gene was significantly correlated with psoriasis [94]. Table 2 summarizes the mechanisms underlying the effects of CASZ1 on various diseases.

**Table 2:** Potential role of CASZ1 in diseases

	Disease types	Gene expression/mutation	Proposed mechanism	Refs.
Cancers	Neuroblastoma	Down		
	Hepatocellular carcinoma	Down	Regulates the MAPK/ERK pathway and MMP2/MMP9 expression, inhibiting abnormal tumor cell proliferation	[74]
	Colorectal cancer Esophageal cancer Lung adenocarcinoma Clear cell renal cell carcinoma	Down	A new prognostic indicator	[17–19, 75]
	Embryonal rhabdomyosarcoma	Down	RAS-MEK signaling or genetic alterations	[76]
	Glioma	Up	Regulates target gene *p75NTR* transcription	[20]
	Epithelial ovarian cancer	Up	Promotes the epithelial–mesenchymal transition of EOC cells	[21]
	Lung adenocarcinoma	Up	Promotes cell migration and invasion by driving ITGAV expression	[78]
Other diseases	Ventricular septal defects (VSD)	Missense mutation p. L38p	–	[22]
	Dilated cardiomyopathy (DCM)	Nonsense mutation p.K351X and c.3781del (p.(Trp1261GlyfsTer29)) frameshift mutation		[23] [25]
	Left ventricular noncompaction cardiomyopathy (LVNC)	p.Val815Profs*14 heterozygous frameshift variant		[24]
	Hypertension	Unknown	Interacts with MR and the Mi-2/NuRD complex to inhibit ENaCα and SGK expression	[81, 83]
	Osteoarthritis	Unknown	Inhibits chondrocyte apoptosis and inflammatory response	[27]
	Immune inflammation	Unknown	Orchestrates T helper (Th) cell differentiation	[87]
	Fasting plasma glucose (FPG)	DNA methylation variants	–	[88]
	Stroke	CASZ1 genetic variants (rs4845941 and rs778228)	–	[90]

## Conclusions

Since its discovery in the early 1990s, the TF CASZ1 has been found to play a crucial role in neural, cardiac, and cardiovascular development. Its utility extends to elucidating the cellular and molecular mechanisms underlying the diversification and subsequent differentiation of neural stem/progenitor cells and cardiomyocytes. In diseases marked by cardiac developmental anomalies, such as DCM, VSD, and LVNC, the loss of CASZ1 expression or genetic mutations can contribute to aberrant cardiac morphogenesis. Notably, the methylation of CASZ1 serves as a regulatory element associated with mortality in patients with cardiovascular disease, and it is reported hypomethylated in Chinese patients with hypertensive cerebral infarction. Furthermore, CASZ1 holds clinical importance in the context of cartilage degeneration and autoimmune inflammation.

CASZ1 plays dual roles in cancer, functioning both as a tumor suppressor and promoter, thereby influencing cancer cell metastasis. The loss of CASZ1 expression or its mutational inactivation is associated with diseases or cancers linked to developmental defects. Moreover, the epigenetic silencing of CASZ1 is linked to poor prognosis in NB. Conversely, CASZ1 is downregulated in hepatocellular carcinoma, colorectal cancer, esophageal cancer, lung adenocarcinoma, and clear cell renal cell carcinoma, and its decreased expression is linked to patient prognosis, with this diminished expression serving as a potential prognostic indicator. Conversely, CASZ1 is highly expressed in glioma and EOC, suggesting a tissue- and tumor-specific expression pattern. The exploration of the potential of CASZ1 as a cancer biomarker for diagnosis and prognosis involves the evaluation of its prognostic value in tumor cells.

In conclusion, as a tumor suppressor gene of neuroblastoma, CASZ1 is involved in the progression of NB through cell cycle regulation and is associated with poor prognosis. Low expression of CASZ1 in hepatocellular carcinoma, colorectal cancer, esophageal cancer, lung adenocarcinoma and clear cell renal cell carcinoma is associated with poor prognosis, while high expression of CASZ1 in glioma, EOC and lung cancer promotes cancer progression. These studies suggest that CASZ1 can be used as a diagnostic and prognostic indicator or as a therapeutic target for cancer. In addition, CASZ1 loss-of-function mutations or mutations at SNPs loci are associated with susceptibility to human diseases, which is of great significance for personalized prevention and treatment of diseases. Therefore, it will be intriguing to investigate the regulatory mechanisms of CASZ1 itself and to identify additional transcriptional targets that could drive development of innovative therapeutic strategies for disease.

## Data Availability

Not applicable.

## Abbreviations

CASZ1: Castor zinc finger 1
NB: Neuroblastoma
EOC: Epithelial ovarian cancer
ZnF: Zinc finger
NLS: Nuclear localization signals
NES: Nuclear export signals
CHD5: Congenital heart disease 5 protein
CID: CHD5-interacting domain
CNS: Central nervous system
RPCs: Retinal progenitor cells
dI1: Dorsal interneuron 1
dILB: Dorsal late-born excitatory
PRC: Polycomb repressive complex
NuRD: Nucleosome remodeling and deacetylase
VSD: Ventricular septal defects
ECs: Endothelial cells
GWAS: Genome-wide association studies
Egfl7: Epidermal growth factor-like domain 7
CRC: Core regulatory circuit
DCM: Dilated cardiomyopathy
LVNC: Left ventricular noncompaction cardiomyopathy
BP: Blood pressure
MR: Mineralocorticoid receptor
